# Vasopressin surrogate marker copeptin as a potential novel endocrine biomarker for antidepressant treatment response in major depression: A pilot study

**DOI:** 10.1192/j.eurpsy.2021.1213

**Published:** 2021-08-13

**Authors:** A. Agorastos, A. Sommer, K. Wiedemann, C. Demiralay

**Affiliations:** 1 Ii. Dept. Of Psychiatry, Aristotle University of Thessaloniki, Thessaloniki, Greece; 2 Psychiatry & Psychotherapy, University Medical Centre Hamburg-Eppendorf, Hamburg, Germany

**Keywords:** biomarker, hypothalamus-pituitary-adrenal (HPA) axis, antidepressant response, Depression

## Abstract

**Introduction:**

Major depressive disorder (MDD) constitutes the leading cause of disability worldwide. Although efficacious antidepressant pharmacotherapies exist for MDD, only about 40-60% of the patients respond to initial treatment. However, there is still a lack of robustly established and applicable biomarkers for antidepressant response in everyday clinical practice.

**Objectives:**

This study targets the assessment of the vasopressin (AVP) surrogate marker Copeptin (CoP), as a potential peripheral hypothalamic-level biomarker of antidepressant treatment response in MDD.

**Methods:**

We measured baseline and dynamic levels of plasma CoP along with plasma ACTH and cortisol (CORT) in drug-naive outpatients with MDD before and after overnight manipulation of the hypothalamic-pituitary-adrenal (HPA) axis [i.e., stimulation (metyrapone) and suppression (dexamethasone)] on three consecutive days and their association with treatment response to 4 weeks of escitalopram treatment.

**Results:**

Our findings suggest significantly higher baseline and post-metyrapone plasma CoP levels in future non-responders, a statistically significant invert association between baseline CoP levels and probability of treatment response and a potential baseline plasma CoP cut-off level of above 2.9 pmol/L for future non-response screening. Baseline and dynamic plasma ACTH and CORT levels showed no association with treatment response.

**Conclusions:**

This pilot study provide first evidence in humans that CoP may represent a novel, clinically easily applicable, endocrine biomarker of antidepressant response, based on a single-measurement, cut-off level. These findings, underline the role of the vasopressinergic system in the pathophysiology of MDD and may represent a significant new tool in the clinical and biological phenotyping of MDD enhancing individual-tailored therapies.

